# Molecular Mechanisms Involved in HCC Recurrence after Direct-Acting Antiviral Therapy

**DOI:** 10.3390/ijms20010049

**Published:** 2018-12-22

**Authors:** Rosanna Villani, Gianluigi Vendemiale, Gaetano Serviddio

**Affiliations:** C.U.R.E. University Centre for Liver Disease Research and Treatment, Department of Medical and Surgical Sciences, Institute of Internal Medicine, University of Foggia, 71122 Foggia, Italy; rosanna.villani@unifg.it (R.V.); gianluigi.vendemiale@unifg.it (G.V.)

**Keywords:** direct-acting antivirals, hepatocellular carcinoma

## Abstract

Chronic hepatitis C is associated with a high risk of developing hepatocellular carcinoma (HCC) because of a direct effect of the Hepatitis C Virus (HCV) proteins and an indirect oncogenic effect of chronic inflammation and impaired immune response. The treatment of chronic hepatitis C markedly reduces all-cause mortality; in fact, interferon-based treatment has shown a reduction of HCC incidence of more than 70%. The recent introduction of the highly effective direct-acting antivirals (DAAs) has completely changed the scenario of chronic hepatitis C (CHC) with rates of HCV cure over 90%. However, an unexpectedly high incidence of HCC recurrence was observed in patients after DAA treatment (27% versus 0.4–2% in patients who received interferon treatment). The mechanism that underlies the high rate of tumor relapse is currently unknown and is one of the main issues in hepatology. We reviewed the possible mechanisms involved in HCC recurrence after DAA treatment.

## 1. Introduction

Chronic viral hepatitis is a well-recognized risk factor for end-stage liver disease and liver cancer [1]. Among primary liver cancers, hepatocellular carcinoma (HCC) accounts for 70% to 85% of cases [2] and it is currently the fifth most common cancer [3] and the third leading cause of cancer mortality in the world [4].

In most cases, cirrhosis promotes hepatocyte regeneration and precedes HCC occurrence [5,6].

Compared with other causes of cirrhosis, chronic hepatitis C is associated with a higher risk of developing HCC [5,7] because the Hepatitis C Virus (HCV) owns a direct oncogenic effect; moreover, chronic inflammation, impairment of immune response, cellular senescence, and proliferation indirectly depend on HCV disease [8,9,10].

Direct oncogenic effects of HCV are related to the cellular expression of viral proteins localized in the cytosol, lipid droplets, endoplasmic reticulum, mitochondria, and nuclei, affecting a variety of cellular functions [10].

Overexpression of HCV proteins, e.g., core, NS3, and NS5A, promotes cellular proliferation, transformation and tumor formation in mice, suggesting a direct effect in activating oncogenic pathways [10,11,12,13,14]. The core protein modulates p53 regulatory activity and directly influences the p53-related p73 protein [15,16] whereas NS3 and NS5A inhibit p53 [17,18]. NS5A promotes evasion from apoptosis by caspase-3 inhibition [19] and inhibits tumor necrosis factor-alpha (TNFα) mediated apoptosis [20], whereas NS5B inhibits the retinoblastoma-associated protein (RB1), which is involved in controlling cellular proliferation and apoptosis by regulating transcription factors [21].

Moreover, viral proteins indirectly regulate innate immune pathways: NS3 suppresses innate immunity by cleavage of the mitochondrial antiviral signaling protein (MAVS), a pivotal antiviral protein involved in interferon induction [22]; the binding of the hepatitis C virus envelope protein E2 to CD81 causes inhibition of natural killer (NK) cells contributing to immune evasion [23]; HCV core protein inhibits hepatocyte senescence, a physiological process providing a barrier to tumorigenesis [24].

HCV-induced HCC is, therefore, a model of chronic inflammation-driven tumor, where a complex interaction between viruses and hepatocytes occurs, promoting hepatocarcinogenesis [10].

The cure of HCV infection defined as the absence of circulating HCV RNA at least 12 weeks after treatment completion (sustained virologic response—SVR), is associated with a marked reduction of the all-cause mortality [25] and particularly, with a reduction of more than 70% of HCC [26]. Compared with interferon-based treatments, direct-acting antivirals (DAAs) achieve SVR rates in over 90% of patients, irrespective of fibrosis stage [27,28,29] and this may significantly improve the natural history of HCV infection. Therefore, DAAs are currently the most promising strategy for reducing the future burden of HCC [30,31]. However, several recent reports have raised concerns about DAA treatment because a higher incidence of HCC recurrence has been observed in patients during and after antiviral treatment [32,33,34,35,36,37,38,39]. These studies have also reported an unexpectedly high incidence of de novo HCC in addition to a particularly aggressive behavior of HCC relapse. A recurrence rate up to 27% (versus 0.4–2% in patients with SVR after interferon treatment) [36,40] has sparked debate on a possible role of DAAs on hepatocellular carcinoma progression and recurrence. Nonetheless, given the lack of robust evidence of a drug-related effect, many authors have investigated all factors potentially involved in promoting liver cancer during or after antiviral treatment.

**Figure 1:**
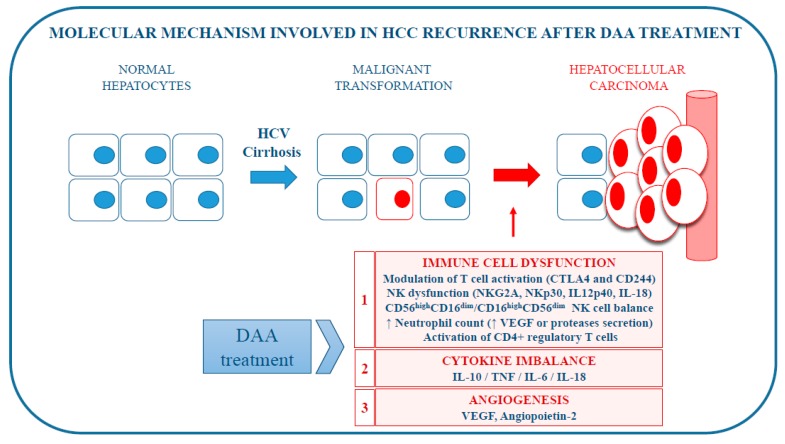
Molecular mechanisms potentially involved in hepatocellular carcinoma (HCC) recurrence after direct-acting antiviral (DAA) treatment for chronic HCV infection.

We reviewed the recent literature that might explain the HCC recurrence after DAA treatment for chronic HCV infection (Table 1 and Figure 1).

## 2. Immune Cell Dysfunction during Chronic HCV Infection

**Figure 2:**
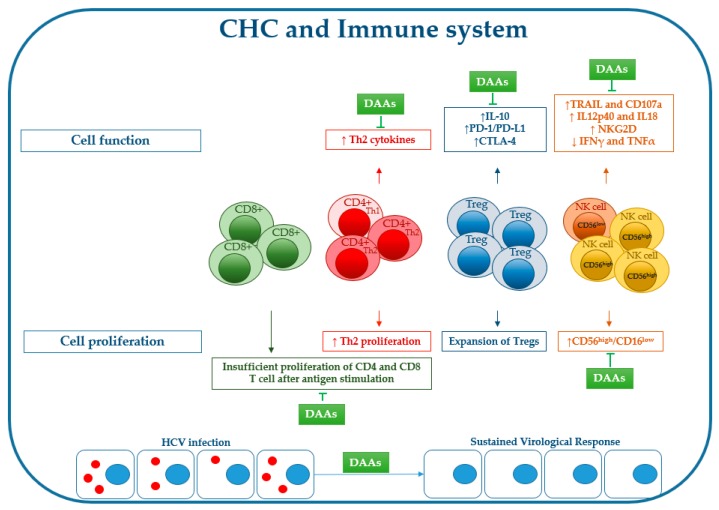
Effects of chronic HCV infection on immune cell proliferation/function and effect of DAA treatment on recovery of virus-induced immune changes (for explanations, see the main text).

The immune system plays a key role both in viral clearance during acute infection and in liver injury during chronic C hepatitis and, because of the lack of a cytopathic effect [53], both innate and adaptive immune responses are necessary to achieve a full recovery from viral infection [54]. However, the interaction between virus and immune system is contradictory, as HCV induces T cell exhaustion and, at the same time, it can stimulate autoantibody production [55] or cause autoimmune diseases [56] (Figure 2).

HCV infection is a leading cause of chronic hepatitis and liver cirrhosis, since in 70–80% of infected individuals, the virus persists after an acute phase without a spontaneous recovery [57]. Viral elimination during acute infection involves both a rapid induction of innate response and a delayed induction of adaptive immune responses.

Innate immune response is the first defense against viral infections and is maintained by interferons (IFNs) that are also able to regulate the cellular components of innate immunity, such as NK cells [58]. After viral infection and endosomal-mediated degradation of viral nucleic acids, macrophages, dendritic cells, NK, and antigen-specific T cells (CD4^+^ Th1 and CD8^+^ cytotoxic T lymphocytes) expose viral antigens to toll-like receptors (TLRs), triggering a nonspecific antiviral response, and induce the transcription of hundreds of genes, which are distinct for different IFNs and target cell types [57].

In contrast to the innate immune response induced within hours to days after infection, adaptive immune responses (humoral antibody and T cells responses) become detectable not before 6–8 weeks after viral infection [59,60,61,62,63,64]. Antibodies blocking virus binding, entry or uncoating were found in most patients with spontaneous viral clearance [65] and, similarly, a long lasting CD4^+^ and CD8^+^ T cell response targeting multiple viral epitopes persists after resolution of acute viral infection, suggesting a major role of adaptive immunity in hepatitis C infection [57,60,66,67,68,69,70,71].

In chronic hepatitis C, HCV typically escapes both innate and adaptive responses [57] thanks to the release of several factors promoting viral immune evasion and globally impairing viral clearance [72], a complex immune mechanism named “T cell exhaustion”, typically characterized by CD4^+^ T cell and CD8^+^ T cell dysfunction together with impaired cytokine production and lack of response to antigen stimulation [73,74].

In acute self-limiting HCV infection, circulating helper T-cells mostly produce IFNα, suggesting Th1 predominance, whereas during chronic infection, a limited Th1-response and a prevalent Th2 response has been reported [75,76,77].

A critical balance between initiation and downregulation of the immune response is required for immune homeostasis and chronic inflammation and autoimmunity may result from dysfunctions of immune resolution [78].

Treatment with anti-interleukin-10 monoclonal antibody enhances IFNα production in HCV patients [79] and a long-term treatment with recombinant IL-10 reduces inflammation in the same patients, suggesting a pivotal role of immunosuppressive cytokine IL-10 in antiviral response and Th1/Th2 balance [75].

Experimental data have shown that a significant decline in CD4^+^/CD25^+^ regulatory cells (also called Treg), typically involved in the maintenance of self-tolerance and suppression of self-reactive lymphocytes [80], is needed to induce suppression of CD8^+^ T cell proliferation and IFN-α production [81,82]. Tregs are involved in the long-term maintenance of memory T cells, ensuring protective immunity, and in controlling the intensity of T cell immune responses, even though this condition allows for inflammation to persist [80]. The presence of CD4^+^/CD25^+^ depends, almost partially, on the production of immunoregulatory cytokines [75]. In contrast to natural Treg cells, which develop in the thymus and play an important role in the maintenance of self-tolerance and immune homeostasis, induced Treg cells develop from nonregulatory T cells in the periphery [83]. Distinct subsets of induced regulatory T cells have been identified, and, among those, Treg cells secreting IL-10 play a pivotal role in viral persistence [83]. During chronic hepatitis C, effector T cells become exhausted and show reduced antiviral activity, while Tregs progressively infiltrate the liver [84]. A higher frequency of Treg in HCV-infected patients was reported by several authors [85,86]. Similarly, Cabrera et al. reported a positive correlation between Treg frequency and HCV RNA and an inverse relation with histologic inflammatory scores, confirming a significant role of regulatory T cells in viral persistence [76].

Therefore, during chronic hepatitis C, effector T cells become exhausted over time and show an impaired antiviral activity while CD4^+^ regulatory T cells (Tregs) accumulate in the liver [84]. Tregs are mainly regulated by interleukin (IL)-2 receptor α chain CD25 and Foxp3 [87]; once activated, Tregs induce expression of programmed cell death protein 1 (PD-1), programmed death-ligand 1 (PD-L1), contact-dependent regulatory molecules such as cytotoxic T-lymphocyte associated protein 4 (CTLA-4) and ultimately cytokines such as IL-10 or transforming growth factor (TGF-β) [48,88,89]. Because of their extensive role in modifying multiple immune functions, such as T cell responses and natural killer (NK) cells function [48], Tregs are considered to be involved in immune surveillance of tumors.

Natural killer (NK) cells are the prototype of innate lymphoid cells with potent cytolytic function that provide viral and immune surveillance against cancer [90]; two subsets of NK cells are commonly recognized: CD56^high^/CD16^low^, which mainly produce chemokines and cytokines, and CD56^low^/CD16^high^, a phenotype with prevalent cytotoxic activity [91]. Generally, NK cells are an important part of the interferon-responsive innate population in the liver (30% of lymphocytes) in comparison to the blood (5–20%), and the percentage increases in viral hepatitis [92,93]. During chronic viral infection, they are permanently activated and polarized toward cytotoxicity with deficient IFNγ secretion because of chronic exposure to endogenous IFNα [94,95]. The result is a “functional dichotomy” presenting with enhanced cytolytic activity and failure to produce IFNγ and TNFα, with consequent inability to eradicate the virus [96]. A possible involvement of NK cells in immune surveillance of tumors might be considered [97].

## 3. DAAs Are Able to Modulate Immune Cell Response

Initial data on T cell function during and after direct-acting antiviral treatment were published in 2014 [98]. The authors analyzed virus-specific CD8^+^ T cells obtained from 51 previously untreated and chronically infected patients undergoing antiviral treatment with a combination of faldaprevir (a protease inhibitor) and deleobuvir (a non-nucleoside polymerase inhibitor) and observed a significant increase in the frequency of HCV-specific CD8^+^ T cells within 4 weeks of therapy in patients obtaining SVR, whereas no changes were observed in patients with treatment failure. This result indicated that the immune dysfunction induced by HCV infection was reversible and that even short therapy with novel DAAs was able to restore immune capacity, whereas the same effect has never been observed in patients treated with interferons [41,97,99,100].

Taken together, these first reports suggested that drugs targeted on HCV replication were able to modulate the immune system by changing T cell balance.

Several authors have addressed the role of NKs in chronic HCV patients during DAA therapy [93]. Before treatment, NK cells showed increased degranulation (studied by the expression of CD107a and tumor necrosis factor-related apoptosis-inducing ligand—TRAIL) and decreased production of cytokins such as IFNγ and TNFα; during treatment, the authors observed a decrease of NK cell-activating receptor expression together with decreased IFNγ production followed by normalization of degranulation. Restoration of immune function induced by DAAs persisted up to the end of treatment and maintained even after suggesting that antiviral treatment is also immunologically effective [96].

These first observations were very impressive, since they did not occur during interferon therapy, where an increase in cytotoxic NK cell activity observed soon after starting therapy lasted only a few hours before leaving a refractory state [101].

In addition, Burchill et al. analyzed the composition of the memory CD4^+^ and CD8^+^ lymphocyte compartment on peripheral blood from nineteen chronic hepatitis C (CHC) patients treated with DAAs. After the rapid eradication of the virus, the frequency of NK cells was unchanged, whereas a significant reduction of the expression of PD1 on the HCV-specific T cells was found, suggesting a partial restoration in the functional capacity of HCV-specific T cells [42].

NK cell frequencies similar to healthy controls were also shown after 12 weeks of interferon-free treatment, adding new reports on the effect of viral load decline on the NK cell compartment. The frequencies of CD56_high_ NK cells (NK_bright_) were typically higher in CHC, whereas CD56_low_ NK cell (NK_dim_) frequencies were lower compared to healthy controls. After treatment with DAAs, NK cell subgroups returned to healthy control levels [43]. Cytokines involved in NK cell activation (IL-12p40 and IL-18), typically higher during chronic hepatitis C, normalized after DAA treatment [43]; the same observation was reported after interferon treatment [102]. Very interestingly, Chu et al. have reported that the pretreatment frequency of CD56^high^/CD16^dim^ NK cells was significantly higher and that of CD16^high^/CD56^dim^ NK cells was significantly lower in patients developing HCC after DAA treatment, showing a significant imbalance in NK cell subgroups. Moreover, the expression of NKG2D was significantly decreased during treatment, even more so in patients who developed HCC [45].

Several studies have reported a decrease of frequencies and activation of monocytes during DAA treatment [46,47], and other reports have suggested the involvement of neutrophils in the vascular endothelial growth factor (VEGF) and proteases secretion that may play a role in cancer cells spreading [44].

All these findings provide a possible rationale to support immune modification induced by DAA treatment, but further studies are needed to clarify the underlying molecular mechanism.

## 4. Cytokine Network Imbalance during Chronic C Hepatitis: Effect of DAAs

Cytokines are low-molecular-weight proteins mainly secreted by macrophages and lymphocytes and involved in cell-to-cell communication [103,104]; they also regulate proliferation, differentiation, migration, and death of immune cells [103].

A typical cytokine pattern of activated T-cell response has been described in chronic HCV patients, with an elevated level of serum IL-2, IL-4, TNFα other than IFN-α and IL-10 [75].

IL-10 is produced by several immune cells, including T and B cells, DC, and macrophages [105], and plays a key role as immunoregulatory molecule [72]; it suppresses antigen-presenting cells and T cell by inhibition of pro-inflammatory cytokine and chemokine production and, finally, by inhibition of costimulation and MHC class II expression [106,107,108]; moreover, viral persistence is associated with a high level of circulating DC-produced IL-10 and activation of PD-1/PD-ligand1 pathway [109,110,111,112,113,114]. In later stages, both NK and CD4^+^ T cells become the main IL-10 producers and play a pivotal role in the regulation of immune response [112,115].

A specific subset of T-cells also produces IL-10, named Tr1 [116], distinct from thymic-derived and naturally occurring CD25^+^Foxp3^+^ Tregs [117]. Tr1 have been found in patients with chronic HCV infection [118,119,120] and a subset of HCV-specific IL-10^+^ CD8^+^ Tr1 has been also described in the liver of such patients [121,122,123].

Since cytokines play a central role in the cross-talk among immune cells during chronic inflammation, some authors have suggested that modification in the circulating level of several cytokines, particularly TNFα, IL-6, and IL-10, may contribute to cancer promotion and progression [124,125,126]. TNFα is one of the cytokines more studied in cancer and has been correlated to chronic lymphocytic leukemia, Barret’s adenocarcinoma, prostate cancer, breast cancer, and cervical cancer [127,128,129,130].

A recent study has shown that, independently from HCV–RNA drop, TNFα remained stable or even higher in those developing HCC as compared to CHC who did not develop HCC [50], but conclusive data are lacking.

IL-6 is another cytokine which plays a significant role in the acute phase response and shows a pro-tumorigenic effect [103]; indeed, specific circulating levels are crucial for hepatocyte homeostasis because of powerful mitogen activity [131]. However, persistent activation of the IL-6 pathway in the liver is associated with the development of liver tumors [131] through a mechanism of hypermethylation of tumor suppressor genes. Very interestingly, serum IL-6 level has been reported to be increased in patients treated with DAAs and developing HCC recurrence [50].

Few data are available on the IL-18 circulating level during DAA treatment. It is considered a pro-inflammatory cytokine, synthesized and secreted by monocytes/macrophages and Kupffer cells. It activates nuclear factor (NF)-κB, which in turn activates cell proliferation, cycle progression, overexpression of angiogenic genes, and apoptosis inhibition [132,133,134]. IL-18 is upregulated in HCV-infected patients and its receptor is commonly expressed in HCC cells.

Two studies have reported a rapid decline of serum IL-18 early after starting DAA treatment [43,51] that was not confirmed by others [46]. Therefore, more data are needed to support a possible involvement of IL-18 in molecular pathway of HCC recurrence.

## 5. Potential Effect of DAAs in the Modulation of Angiogenesis Signaling

Angiogenesis is a dynamic process in which new vascular structures develop from preexisting vessels [135]. Key regulators of this process are hypoxia and inflammation, which stimulate the release of the hypoxia inducing factor (HIF), angiogenic cytokines, and growth factors from different cell types, including endothelial cells (ECs), monocytes, platelets, and smooth muscle, as well as tumor cells, and promote endothelial cell proliferation and stabilization of neovessels [135,136,137,138].

The most relevant angiogenic factor is VEGF (vascular endothelial growth factor), which has a key role in angiogenesis during inflammation and new vessel formation in tumors [139,140]. It is a potent growth factor produced mainly by tumor cells, macrophages, and platelets, and it is a highly specific mitogen for endothelial cells [141,142]. VEGF is activated by oncogenes and several cytokines [142] and functions as a cytokine promoting endothelial cell proliferation, vessel permeability, disruption of tight junction, and finally, the proliferative activity and the neoangiogenic potential of the tumor [143,144,145]. The effects of VEGF are mainly mediated in endothelial cells via VEGFR2. The activation of VEGFR2 receptor induces dilation of vessels, which also became leaky in response to VEGF signaling.

Both VEGF and Angiopoietin-2 (Ang2), a growth factor specific for the vascular endothelium and expressed during vascular remodeling in tumors [146,147], are involved in the dissolution of the vascular basement membrane and assembly into vascular networks [139] and serum concentration have been investigated to assess possible implications in liver diseases.

Hepatic neoangiogenesis has been described in liver diseases, such as viral hepatitis, cirrhosis, and HCC [148,149], and hepatitis C virus infection was found to induce production of TGF beta [137] and stabilization of HIF, resulting in the release of angiogenic cytokines [150] and proliferation of human endothelial cells [135,151].

Among the factors contributing to liver damage during chronic hepatitis C, angiogenesis seems to play a pivotal role [138,149,152] and the degree of microvessel density is increased in HCV-positive patients [153].

The serum concentrations of VEGF and Ang2 are also increased in patients with HCC compared to patients without liver cancer [154].

We observed a rapid increase of VEGF level in patients treated with all DAA regimens. After 4 weeks of treatment, in comparison to baseline, serum VEGF remained higher until the end of treatment and returned to baseline concentration after stopping the therapy [49]. Our findings were recently confirmed by Faillaci et al., who analyzed serum liver angiopoietin-2 and VEGF levels in 242 DAA-treated patients and found a DAA-mediated increase of VEGF and angiopoietin-2 supported the increased risk of HCC recurrence/occurrence during antiviral treatment [52].

Debes et al. confirmed a potential role of VEGF in risk of hepatocellular carcinoma, but they showed an alteration of baseline serum VEGF concentration in patients who developed HCC de novo, supporting the possible pre-existing increased VEGF level without a direct role of DAAs in liver cancer [50]. Further studies are required to definitively clarify the role of DAAs on tumor recurrence.

## 6. Conclusions

DAA treatment has completely changed the natural history of chronic hepatitis C, with the rate of cure over 90%. However, some authors have reported a worrying increase in HCC recurrence. Among the pathophysiological hypotheses, the most appealing one suggests that DAAs induce dramatic HCV clearance that in turn may induce immune cell alteration, imbalance of cytokine network, and angiogenesis that could explain, almost in part, the HCC recurrence observed after DAAs. Data are not conclusive, and the final word remains to be said.

## Figures and Tables

**Table 1:** Summary of potential mechanisms involved in HCC recurrence.

Hypothesis	Molecular Mechanism	Author	Ref.
Immune cell dysfunction	Modulation of T cell activation	Meissner et al., 2014	[41]
	Reduced NK cell activation	Burchill et al., 2015	[42]
	Downregulation of NKG2A receptor	Spaan et al., 2016	[43]
	Declined IL12p40, IL-18 serum level	Spaan et al., 2016	[43]
	Modulation of differential white blood cell count	Casadei Gardini et al., 2018	[44]
	Imbalance in NK cell subgroups	Chu et al., 2017	[45]
	Decreased NKG2D	Chu et al., 2017	[45]
	Decreased frequencies of NK cells	Ning et al., 2017;	[46]
		Meissner et al., 2017	[47]
	Immunosuppressive Tregs function	Langhans et al., 2017	[48]
Change in immune cytokine network	Rapid reduction of IL-10 serum level	Villani et al., 2016	[49]
	Increased TNFα secretion	Debes et al., 2018	[50]
	Change in IL-6 serum level	Debes et al., 2018	[50]
	Change in IL-18 serum level	Spaan et al., 2016;	[43]
		Ning et al., 2017;	[46]
		Carlin et al., 2015	[51]
Activation of angiogenesis	Increase of VEGF serum level	Villani et al., 2016;	[49]
		Faillaci et al., 2018	[52]
	Increase of angiopoietin-2 serum level	Faillaci et al., 2018	[52]

## Abbreviations

HCC	hepatocellular carcinoma
CHC	chronic hepatitis C
NS3	nonstructural protein 3
NS5A	nonstructural protein 5A
TNFα	tumor necrosis factor-alpha
NS5B	nonstructural protein 5B
RB1	retinoblastoma-associated protein
MAVS	mitochondrial antiviral signaling protei
NK	natural killer
SVR	sustained virologic response
DAAs	direct-acting antivirals
IL12p40	p40 subunit of IL12
Treg	regulatory T cell
VEGF	vascular endothelial growth factor
IFN	interferon
TLR	toll-like receptor
IFNα	interferon alpha
TGFβ	transforming growth factor beta
PD-L1	programmed death-ligand1
CTLA-4	cytotoxic Tl-lymphocyte associated protein 4
IFNγ	interferon gamma
TRAIL	tumor necrosis factor-related apoptosis-inducing ligand
PD-1	programmed death 1
Ang-2	angiopoietin
VEGFR2	vascular endothelial growth factor receptor 2
IL-2	interlukin 2
IL-4	interlukin 4
IL-6	interleukin 6
IL-10	interleukin 10
IL-18	interleukin 18
